# Retinal microvasculature and cerebral hemodynamics in patients with internal carotid artery stenosis

**DOI:** 10.1186/s12883-022-02908-7

**Published:** 2022-10-13

**Authors:** Junfeng Liu, Jincheng Wan, William Robert Kwapong, Wendan Tao, Chen Ye, Ming Liu, Bo Wu

**Affiliations:** 1 Department of Neurology, West China Hospital, Sichuan University, No. 37, GuoXue Xiang, Chengdu, Sichuan Province 610041 People’s Republic of China; 2 Department of Neurology, Minda Hospital of Hubei Minzu University, Enshi, 445000 Hubei Province China

**Keywords:** Internal carotid artery stenosis, Swept-source optical coherence tomography angiography, Retinal microvasculature, Computed tomography perfusion, Cerebral hemodynamics

## Abstract

**Purpose:**

To investigate the relationship between retinal microvasculature and cerebral hemodynamics in patients with internal carotid artery (ICA) stenosis.

**Methods:**

Patients with unilateral moderate or severe ICA stenosis(≥50%) from West China hospital, Sichuan university were consecutively and prospectively recruited enrolled in the current study. En face angiograms of the superficial vascular complex (SVC), deep vascular complex (DVC), superficial vascular plexus (SVP), intermediate capillary plexus (ICP), and deep capillary plexus (DCP) were generated by automatic segmentation using swept-source optical coherence tomography angiography (SS-OCTA) to assess the retinal microvascular perfusion. The cerebral blood flow perfusion on bilateral middle cerebral artery territories measured at the basal ganglia level was assessed by brain computed tomography perfusion (CTP). CTP data were postprocessed to generate maps of different perfusion parameters including cerebral blood flow (CBF), cerebral blood volume (CBV), time to peak (TTP), mean transit time (MTT) and permeability surface(PS). Relative perfusion parameters (rPS, rCBF, etc.) were calculated as the ratio of the value on the contralateral side to that on the ipsilateral side.

**Results:**

In the final analysis, 31 patients were included, of whom 11 patients had a moderate ICA stenosis (50–69%) and 20 with a severe ICA stenosis(≥70%). A total of 55 eyes were analyzed in the study, 27 eyes from the ipsilateral side (ie, side with stenosis) and 28 eyes from the contralateral side. In the patients with ICA stenosis, there was a strong correlation between the retinal microvascular perfusion of SVC with rCBV(B = 0.45, *p* = 0.03), rCBF(B = 0.26, *p* = 0.02) and rPS(B = 0.45, *p* < 0.001) after adjustment for age, sex and vascular risk factors. Similar correlations were also found between microvasculature in SVP and cerebral perfusion changes. There were no any significant associations of microvascular perfusion in both DVC and DCP with CTP parameters(all *p* > 0.05).

**Conclusions:**

Retinal perfusion changes in superficial vascular layer (SVC and SVP) were correlated with brain hemodynamic compromise in patients with unilateral moderate or severe ICA stenosis(≥50%). Given the limited size of our study, future studies with larger sample size are needed to confirm our findings.

## Introduction

Internal carotid artery(ICA) stenosis or occlusion is associated with a transient ischemic attack (TIA) and ischemic stroke, and the risk increases with increasing severity of the stenosis [1, 2]. In addition, patients with ICA stenosis were also reported to have cognitive deterioration [3], and impaired cerebral hemodynamics may be involved in this [4–6]. Therefore, the availability of biomarkers to identify such high-risk of subgroups who are particularly prone to developing cognitive decline and could benefit from more aggressive treatment strategies [5, 7].

Recent reports have shown that brain CT perfusion (CTP) is a fast and reliable tool for evaluating cerebral perfusion [8] in patients with acute ischemic stroke and ICA stenosis; importantly, the CT perfusion measurements of cerebral blood flow (CBF) were accurate and stable when compared to transcranial doppler(TCD),xenon CT and H2^15^O PET [9–11]. In addition, mean transit time (MTT) is the most sensitive and reproducible CT perfusion parameter to detect brain hemodynamic changes in patients with ICA stenosis [12, 13]. Although the CTP is a useful imaging tool, it is time-consuming, costly, and has some contraindications (some patients are unable to tolerate CTP because of their allergic to contrast agent or renal dysfunction). Therefore, simpler, inexpensive, reproducible tools for assessing cerebral hemodynamics in patients with ICA stenosis would offer the prospect of identifying individuals with a high risk of stroke and may play a role in personalized and tailored treatment.

The retina shares embryologic origin and microvascular characteristics with the brain and is widely regarded as part of the central nervous system [14]. Moreover, the ophthalmic artery, which supplies blood to the retina, is a branch of the internal carotid artery; thus, structural changes in the internal carotid artery may affect the vasculature and structure of the retina. Notably, retinal thickness and microvasculature offer a unique route to assess cerebral microstructural and microvasculatural changes. Several studies [15–18] suggested that ocular manifestations are related to the changes of carotid artery including the severity of carotid stenosis. However, few study has investigated [19] whether retinal microvasculature abnormalities can reflect the cerebral hemodynamic changes in patients with ICA stenosis.

Swept-source optical coherence tomography angiography (SS-OCTA) is an in vivo imaging tool that allows noninvasive, high-resolution examination of the retinal microvasculature. Compared to spectral-domain optical coherence tomography angiography, SS-OCTA tool has a longer wavelength and a faster scan speed which enables a more accurate three-dimensional image of the retinal microvasculature [20]. Automated segmentation techniques make a quantitative assessment of retinal microvasculature a viable proposition. This offers the prospect of retinal imaging contributing to assessing cerebral hemodynamic changes in patients with ICA stenosis.

Therefore, in the study, en face angiograms of the superficial vascular complex (SVC), deep vascular complex (DVC), superficial vascular plexus (SVP), intermediate capillary plexus (ICP), and deep capillary plexus (DCP) were generated by automatic segmentation to assess the retinal microvasculature by SS-OCTA. Then, we assess the relationship between retinal microvasculature, and cerebral perfusion parameters assessed on CTP in patients with ICA stenosis.

## Methods

### Participants

Patients with unilateral symptomatic or asymptomatic carotid stenosis admitted to the department of neurology, West China Hospital, Sichuan university were consecutively and prospectively recruited for our study since 2020 December 1st.

All the participants signed informed consent. The study was approved by the Biomedical Research Ethics Committee of West China Hospital, Sichuan University (2020[922]), and followed the principles of the Declaration of Helsinki.

### Inclusion and exclusion criteria

The inclusion criteria of our participants were as follows: (1) age ≥ 18 years; (2) patients with unilateral internal carotid stenosis≥50% or more as confirmed on CT angiography(CTA) or digital subtraction angiography(DSA); (3) the cerebral perfusion status on the ipsilesional middle cerebral artery(MCA) territory was apparently normal, i.e. comparable to the contralateral side, defined as a relative difference in cerebral blood flow (CBF) ≤ 30% between bilateral MCA territories on CT perfusion (CTP) maps; (4) patients who completed CTP and also could cooperate with SS-OCTA imaging.

Patients were excluded as follows: (1) non-atherosclerotic intracranial stenosis (e.g. Moyamoya disease, vasculitis, or dissection); (2) previous known or evidence of arteriovenous malformation or aneurysm; (3) poor SS-OCTA images with signal quality less than 7 [21]; (4) with other neurological diseases such as Alzheimer’s disease, Parkinson’s disease, and multiple sclerosis; (5) with ophthalmic abnormalities that could potentially impact the structure and microvasculature of the retina such as diabetic retinopathy, pre-existing glaucoma, cataract, age-related macular degeneration, optic neuritis, and significant myopia; (6) diopter > ± 3.0 (may affect visual acuity); or (7) previous interventional or surgical procedures in the ipsilesional extra- or intracranial arteries.

### Data collection

#### Clinical characteristics

Demographic (age and sex), vascular risk factors (hypertension, diabetes, hyperlipidemia, coronary artery disease, smoking, previous TIA, previous stroke and alcohol consumption information) were collected from the medical records.

#### Retinal imaging with swept-source optical coherence tomography angiography (SS-OCTA)

The SS-OCTA examinations were performed by an expert examiner(William Robert Kwapong). With a central wavelength of 1050 nm and a scan rate of 200,000 A-scan per second, the SS-OCTA which contained a swept-source laser was used to image the retina of all participants. The tool was set with an eye-tracking function based on an integrated confocal scanning laser ophthalmoscope to remove eye-motion artifacts. The lateral resolution, axial resolution, and scan depth were 13 μm, 5 μm, and 3 mm respectively.

**Fig. 1:**
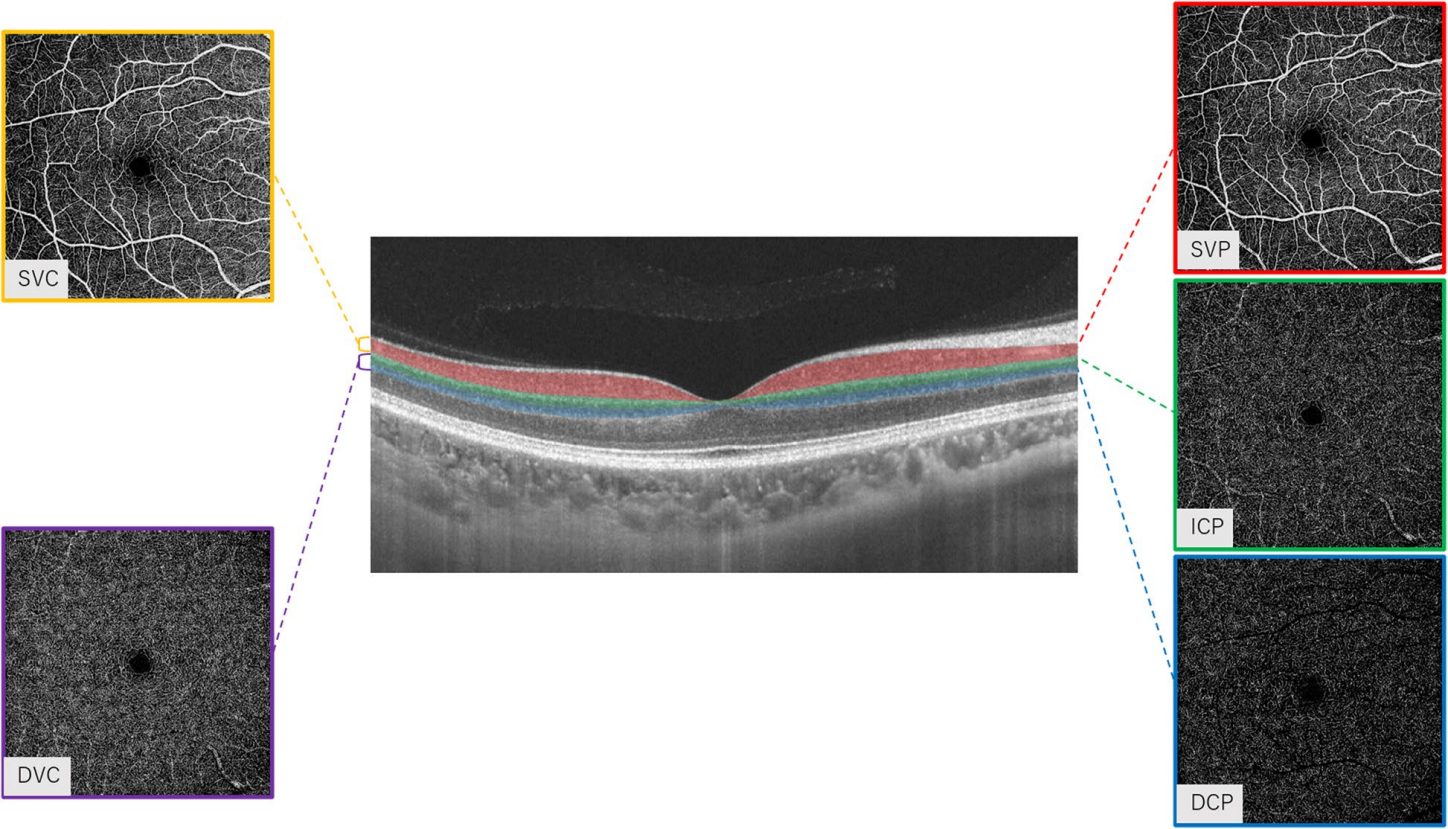
Segmentation of the two macular complexes in the fovea. SVC: superficial vascular complex, DVC: deep vascular complex, SVP: superficial vascular plexus, ICP: intermediate capillary plexus; DCP: deep capillary plexus

OCTA fundus images were obtained with a raster scan protocol of 512 horizontal B-scans that covered an area of 6 mm^2^ centered on the fovea. The proposed OCTA nomenclature segmentation was used in our study. En face angiograms of the superficial vascular complex (SVC), deep vascular complex (DVC), superficial vascular plexus (SVP), intermediate capillary plexus (ICP), and deep capillary plexus (DCP) were generated by automatic segmentation to assess the retinal microvascular perfusion. Segmentation of the SVC and DVC was defined as the inner two-thirds and outer one-third interface of the ganglion cell layer and inner plexiform layer (GCL + IPL) as shown in Fig. 1. The SVP was defined as the microvasculature between the base of the retinal nerve fiber layer (RNFL) to the junction between the inner plexiform layer (IPL) and inner nuclear layer (INL); ICP was defined as the microvasculature between IPL/INL junction to the junction between INL and outer plexiform layer (OPL); DCP was defined as the microvasculature between the INL/OPL junction to 25 μm below the OPL as shown in Fig. 1.Microvascular perfusion was used to assess the microvasculature of the macular plexuses and was defined as the total length of perfused microvasculature per unit area in square millimeters (mm2) in the annulus region of measurement (6 × 6 mm2 around the fovea). A random sample of 20 participants were chosen to evaluate the retinal microvasculature twice in order to assess intra-rater agreement. The intra-rater kappa coefficient was 0.97 for SVC, 0.98 for DVC, 0.93 for SVP, 0.97 for ICP and 0.97 for DCP.

#### Computed tomography perfusion (CTP) imaging protocol and postprocessing

All patients were examined by CTP to evaluate the cerebral blood flow perfusion of patients with carotid artery stenosis. CTP imaging was performed on a 64-slice CT scanner (Discovery CT750 HD; GE Medical Systems, Waukesha, Wisconsin) or a 128-row dual-source CT scanner (Siemens SOMATOM Definition Flash, Siemens Healthcare, Forchheim, Germany) depending on device availability. CT scanning was initiated 5 s (GE) or 5 s (Siemens) after a contrast agent bolus (350 mg/mL Omnipaque followed by a saline flush of 45 mL at 5 mL/s), as follows: Jog mode, 80 kVp/150 mAs (GE) and 80kVp/200mAs (Siemens); 26 cycles for 42 s (GE) and 30 cycles for 45 s (Siemens); and 312 slices (GE)and 128 slices (Siemens). Brain standard reconstruction was performed. The gantry angle was parallel to and above the orbital roof to avoid radiation exposure to the lens.

### CTP postprocessing

The CTP images were analyzed by one experienced neurologist (Junfeng Liu). CTP data were transferred to a workstation (IntelliSpace Portal system, Philips Healthcare) to generate perfusion parameter maps of cerebral blood flow (CBF), cerebral blood volume (CBV), time to peak (TTP), mean transit time (MTT) and permeability surface(PS). The PS was calculated based on Patlak model. Regions of interest (ROIs) were drawn on CTP source images and transferred to corresponding parametric maps. The source images were average images calculated from all phases that could offer accurate anatomical references. Absolute values of CBF, CBV, TTP, MTT and PS on the bilateral MCA territories were measured at the basal ganglia level, by drawing symmetrical regions of interest in the two hemispheres on perfusion maps. Any region of old or new infarct was excluded from such measurement. We measured the basal ganglia level three times; finally, the average value was taken to ensure that the final selected ROI data were reliable and consistent. Relative perfusion parameters (rPS, rCBF, etc.) were calculated as the ratio of the value on the contralateral side to that on the ipsilateral side for each ROI.

### Sample size calculation

The sample size was calculated on the basis of the previous study [22] using the correlation coefficient between retinal microvascular changes and white matter lesions(*r* = 0.40). Assuming a power of 80% and α = 0.05, in a two- tailed test, we will need a sample of 36 individuals at least.

### Statistical analysis

Continuous variables with normal distribution were expressed as mean ± standard deviation (SD), while skewed distribution was as medians and interquartile ranges. Categorical variables are presented as frequencies and percentages. Multivariable linear regression models were used to investigate the association between SS-OCTA parameters and perfusion parameters while adjusting for age, gender, and vascular risk factors. All statistical analyses were conducted using the statistical software R version 3.4.1(http://www.R-project.org). The difference was considered statistically significant if the *P*-value was < 0.05.

### Availability of supporting data

The data that support the results of this study are available from the corresponding author upon reasonable request.

## Results

### Clinical and imaging characteristics

From 1st December,2020 to 30th November,2021, 56 patients with unilateral ICA stenosis were enrolled, of whom 14 patients were excluded due to poor quality of CTP scans, 9 patients were excluded due to the relative difference of CBF between bilateral MCA territories> 30%, and 2 patients were excluded due to poor quality of OCTA images. Thus, 31 patients were analyzed in the current study. The mean age was 64.8 ± 10.7 years, and 87.1% were men.

**Fig. 2:**
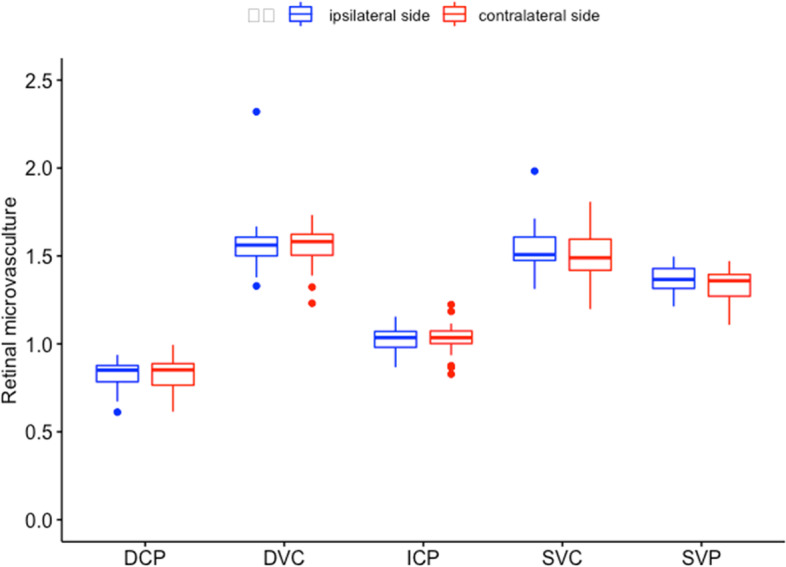
The differences of retinal parameters of eyes on ipsilateral and contralateral side. SVC, superficial vascular complex; DVC, deep vascular complex; SVP, superficial vascular plexus; ICP, intermediate capillary plexus; DCP, deep capillary plexus

The characteristics of the patients are presented in Table 1. Overall, there were 11 (35.5%) patients with moderate ICA stenosis (50–69%) and 20(64.5%) with severe ICA stenosis(≥ 70%). Only 3 patients were admitted with ophthalmic symptoms, but none of them had visual field dysfunction through our physical examination. A total of 55 eyes were included in the study, 27 eyes from the ipsilateral side (ie, the side with stenosis) and 28 eyes from the contralateral side. The retinal microvascular parameters were comparable between ipsilateral and contralateral side (Fig. 2, all *p* > 0.05).

**Table 1:** Baseline characteristics of the 31 ICA stenosis patients

Characteristics	Overall(***n*** = 31)	Moderate stenosis(> 50–69%)(***n*** = 11)	Severe stenosis(≥ 70%)(***n*** = 20)	P
Age, (median [IQR])	64.00 [58.50, 73.00]	68.00 [65.50, 76.50]	62.50 [52.75, 70.50]	0.032
Male, n(%)	27 (87.1)	10 (90.9)	17 (85.0)	0.639
**Vascular risk factors**				
Hypertension, n(%)	19 (61.3)	8 (72.7)	11 (55.0)	0.452
Diabetes mellitus, n(%)	7 (22.6)	4 (36.4)	3 (15.0)	0.21
Hyperlipidemia, n(%)	5 (16.1)	2 (18.2)	3 (15.0)	0.818
Coronary heart disease, n(%)	3 (9.7)	1 (9.1)	2 (10.0)	0.935
Previous TIA, n(%)	4 (12.9)	3 (27.3)	1 (5.0)	0.115
Previous stroke, n(%)	9 (29.0)	3 (27.3)	6 (30.0)	0.873
Smoking, n(%)	21 (67.7)	8 (72.7)	13 (65.0)	0.660
Drinking, n(%)	16 (51.6)	7 (63.6)	9 (45.0)	0.458
**CT perfusion parameters**				
CBV, (median [IQR])	4.31 [3.59, 4.64]	3.86 [3.25, 4.51]	4.37 [3.94, 4.73]	0.173
rCBV, (median [IQR])	1.00 [0.95, 1.03]	1.01 [0.98, 1.08]	0.99 [0.92, 1.02]	0.107
CBF, (median [IQR])	43.29 [37.98, 48.13]	47.55 [41.80, 53.41]	40.78 [35.52, 46.79]	0.029
rCBF, (median [IQR])	1.08 [0.95, 1.21]	1.05 [0.90, 1.11]	1.16 [1.03, 1.28]	0.099
MTT, (median [IQR])	6.55 [5.69, 7.76]	6.07 [5.32, 6.65]	7.00 [5.96, 8.54]	0.037
rMTT, (median [IQR])	0.89 [0.85, 1.04]	1.07 [0.96, 1.07]	0.87 [0.76, 0.93]	0.006
TTP, (median [IQR])	20.90 [18.89, 21.81]	19.33 [18.57, 20.41]	21.53 [19.88, 21.92]	0.063
rTTP, (median [IQR])	0.98 [0.95, 1.00]	1.00 [0.99, 1.02]	0.96 [0.94, 0.98]	0.001
PS, (median [IQR])	14.38 [9.55, 16.67]	11.51 [8.91, 16.27]	14.52 [11.78, 16.93]	0.457
rPS, (median [IQR])	1.01 [0.91, 1.06]	1.04 [0.95, 1.06]	1.00 [0.87, 1.05]	0.409

PS is expressed in mL·100 g^−1^·min^−1^; CBF in mL·min^− 1^·100 g^− 1^; CBV in mL·100 g^− 1^; and MTT and TTP are expressed in seconds

*r* relative, *CBF* cerebral blood flow, *CBV* cerebral blood volume, *MTT* mean transit time, *TTP* time to peak, *PS* permeability surface

### Brain and retinal perfusion parameters between moderate and severe ICA stenosis group

**Fig. 3:**
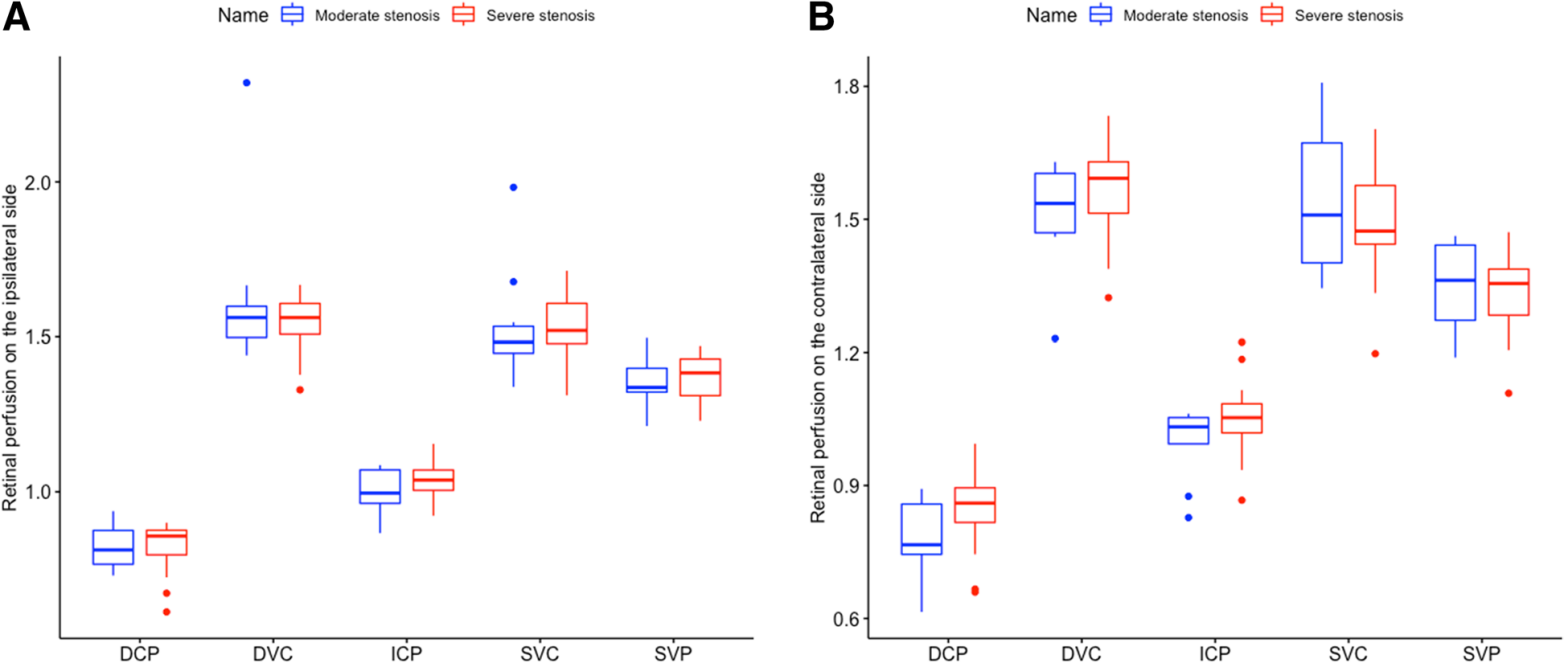
The association between the severity of internal carotid artery stenosis and retinal microvasculature. SVC, superficial vascular complex; DVC, deep vascular complex; SVP, superficial vascular plexus; ICP, intermediate capillary plexus; DCP, deep capillary plexus; ICA, internal carotid artery

Concerning brain perfusion parameters, patients with severe ICA stenosis had significantly higher MTT (median,7.00 vs.6.07, *P* = 0.037) and lower CBF (median,40.78 vs.47.55, *P* = 0.029) values on the ipsilateral side compared to patients with moderate ICA stenosis (Table 1). The other brain perfusion parameters and clinical characteristics were not significantly different among the two groups, except age (Table 1; *P* = 0.032). The retinal microvasculature on the ipsilateral (Fig. 3A) and contralateral side (Fig. 3B; all *p* > 0.05) were both comparable between patients with moderate and severe stenosis.

### Correlation between SS-OCTA parameters and brain perfusion parameters

After adjustment for confounders, the perfusion of SVC showed a significant correlation with rCBV(B = 0.45, *p* = 0.03), rCBF(B = 0.26, *p* = 0.02),rPS(B = 0.45, *p* < 0.001) and PS on the ipsilateral side(B = 0.02, *p* = 0.007). Likewise, the perfusion of SVP was also found to be significantly related to rCBF(B = 0.07, *p* = 0.01),rMTT(B = -0.27, *p* = 0.006) and rPS(B = 0.25, *p* = 0.002).In addition, the perfusion of ICP showed a significant association with rPS(B = 0.14, p = 0.02) in multivariate linear regression analysis. DVC and DCP did not show any significant correlation (*P* > 0.05) with CTP parameters as shown in Table 2.

**Table 2:** Correlation between retinal microstructure and brain perfusion in multivariate linear regression analysis^a^

	SVC			DVC			SVP			ICP			DCP		
	B	SE	P	B	SE	P	B	SE	P	B	SE	P	B	SE	P
**CBV**	−0.03	0.02	0.12	−0.01	0.02	0.75	−0.01	0.01	0.46	0.01	0.01	0.51	−0.02	0.01	0.09
**rCBV**	0.45	0.20	0.03	0.14	0.22	0.52	0.20	0.14	0.16	0.11	**0.11**	0.31	0.10	0.11	0.37
**CBF**	−0.01	0.01	0.80	−0.01	0.01	0.81	−0.01	0.01	0.36	−0.01	0.01	0.94	−0.01	0.01	0.10
**rCBF**	0.26	0.11	0.02	0.08	0.12	0.50	0.19	0.07	0.01	0.09	0.06	0.11	0.10	0.06	0.10
**MTT**	0.01	0.01	0.93	0.01	0.01	0.46	0.01	0.01	0.33	0.01	0.01	0.09	0.01	0.01	0.05
**rMTT**	−0.27	0.14	0.06	−0.02	0.16	0.91	−0.27	0.09	0.006	−0.12	0.07	0.12	−0.12	0.08	0.12
**TTP**	0.02	0.01	0.12	0.01	0.01	0.29	0.01	0.01	0.08	0.01	0.01	0.12	0.01	0.01	0.10
**rTTP**	−0.14	0.46	0.76	−0.32	0.48	0.51	−0.46	0.31	0.14	−0.18	0.23	0.44	−0.24	0.24	0.31
**PS**	0.02	0.01	0.007	−0.01	0.01	0.68	0.01	0.01	0.004	0.01	0.01	0.24	−0.02	−0.03	0.59
**rPS**	0.45	0.11	< 0.001	0.14	0.06	0.66	0.25	0.08	0.002	0.14	0.06	0.02	−0.01	0.07	0.97

^a^adjusting for age, sex, vascular risk factors (hypertension, diabetes, hyperlipidemia, coronary artery disease, smoking, previous TIA, previous stroke and alcohol consumption information) and degree of stenosis

*CBV* cerebral blood volume on the ipsilateral side, *CBF* relative cerebral blood flow on the ipsilateral side, *MTT* relative mean transit time on the ipsilateral side, *TTP* time to peak on the ipsilateral side, *PS* permeability surface on the ipsilateral side, *rCBV* relative cerebral blood volume, *rCBF* relative cerebral blood flow, *rMTT* relative mean transit time, *rTTP* relative time to peak, *rPS* relative permeability surface, *SVC* superficial vascular complex, *DVC* deep vascular complex, *SVP* superficial vascular plexus, *ICP* intermediate capillary plexus, *DCP* deep capillary plexus

## Discussion

Our study shows a novel association of retinal microvascular changes in SVC and SVP with brain perfusion among patients with unilateral carotid stenosis(> 50%). It suggests that retinal microvasculature may reflect brain hemodynamic changes among patients with carotid stenosis. Therefore, OCTA may be used as a potential noninvasive quantitative screening tool for the assessment of cerebral hemodynamic compromise.

The ophthalmic artery is an important branch of ICA, and ICA stenosis can affect structure and microvasculature of the retina [23]. Previous studies reported that patients with carotid stenosis have thinner retinal nerve fiber layer (RNFL) thickness and decreased ocular vessel density than healthy group [16, 19, 24]. However, whether retinal microvascular changes could reflect brain hemodynamics among patients with ICA stenosis remains unknown. To the best knowledge, our current report is the first to show a significant correlation between retinal microvasculature and cerebral microcirculation in patients with ICA stenosis. Given the homology between the retinal and cerebral microvasculature, concomitant cerebral hemodynamic changes in ICA stenosis might extend to the retina, causing changes in the microvascular network. Similarly, reports using different OCTA machines showed patients with ICA stenosis have significantly decreased superficial vasculature densities compared with controls [25, 24]. It suggested the superficial vessels of the retina are sensitive to ischemic injury [24], which are the main blood flow channel and responsible for the arterial circulation of the retina [26–28]. This may help us to understand the significant association of retinal perfusion in superficial vascular layer (SVC and SVP) with brain perfusion changes found in our study.

Previous reports using different imaging modalities have shown that the superficial vessels of the retina are significantly altered in patients with cerebrovascular diseases [29–31] and may be a potential risk indicator of ischemic stroke [32]. Since ICA stenosis causes cerebral hypoperfusion [33], accounting for 30–50% ischemic strokes in Asia [34], it is plausible that changes in the superficial vessels may give clues to the occurrence of ischemic stroke in patients with ICA stenosis. Despite the preliminary nature of the study, our results pave the stone for future extensive investigations on whether the retinal microvascular changes assessed by SS-OCTA could be a potential biomarker to identify brain hemodynamic compromise and help to predict future stroke, TIA and dementia risks in patients with ICA stenosis.

In our study, patients with severe ICA stenosis showed a higher MTT, rMTT, rTTP and lower CBF of ipsilateral side compared to moderate ICA stenosis (Table 1, all *P* < 0.005). It is consistent with other reports that the severity of ICA stenosis is related to cerebral hypoperfusion, which may have a higher risk of ischemic stroke or recurrence [35, 36]. However, our data did not show any significant differences of retinal microvasculature between patients with moderate and severe ICA stenosis (Fig. 3). Moreover, the brain perfusion parameters on the contralateral side were similar between patients with moderate and severe ICA stenosis (data not shown). It may be related to the small sample size included in the study (11 moderate ICA stenosis and 20 severe ICA stenosis). Another explanation might be that moderate and severe ICA stenosis have similar effect on the retinal microvasculature and cerebral microcirculation on the contralateral side. In addition, previous studies reported that patients with carotid artery stenosis had poor retinal microvasculature compared to healthy controls [37–39]. However, few study has investigated the retinal perfusion changes in different severity of ICA stenosis, especially between moderate and severe ICA stenosis. Longitudinal studies with larger sample sizes are needed to investigate this.

Moreover, we did not find any significant differences in retinal microvasculature between ipsilateral and contralateral eyes. During stenosis, ipsilateral circulation may rely on contralateral circulation for support (a compensatory mechanism), which may explain the insignificant difference between the contralateral and ipsilateral retinal microvasculature. Given the small sample size of the subjects in the study, future studies with larger sample sizes are needed.

Our study has several limitations. Firstly, since our study was a cross-sectional study, it is also not certain when the retinal perfusion change occurs, whether the retinal perfusion changes in patients with ICA stenosis could be an indicator for predicting a broad spectrum of neurological disorders such as ischemic stroke, transient ischemic attack, and vascular dementia. In the future, more studies with follow-up data are needed to explore this. Secondly, bias might be caused by the small sample size, such as only 4 female patients and no healthy group were included in our study. Thirdly, brain circulation is related to the collateral status and the duration of stenosis, both of which were not available in the study. Whether they may affect the relationship between retinal microvasculature and brain microcirculation needs more study to investigate.

## Conclusions

In conclusion, our study provides the first evidence that the retinal microvascular changes in superficial vascular layer (SVC and SVP) were correlated with brain hemodynamics in patients with moderate and severe ICA stenosis (≥50%). The retinal microvascular changes assessed by SS-OCTA could be potential biomarkers to identify brain hemodynamic compromise in ICA stenosis. Future studies with larger sample size are needed to confirm our findings.

## Data Availability

The datasets generated during and analyzed during the current study are not publicly available due to patient privacy and ownership issues but are available from the corresponding author on reasonable request.
